# Predictive Model for Critical Illness Infection in Hospitalized Children with RSV Infection: A Retrospective Study

**DOI:** 10.3390/diagnostics16111701

**Published:** 2026-05-31

**Authors:** Xingfeng Cheng, Sha Wei, Jinquan Xia, Kai Zhou, Dan Sun

**Affiliations:** 1 Intensive Care Unit, Wuhan Children’s Hospital (Wuhan Maternal and Child Healthcare Hospital), Tongji Medical College, Huazhong University of Science & Technology, Wuhan 430074, China; 2 Department of Central Laboratory, Shenzhen People’s Hospital (The Second Clinical Medical College), Jinan University, The First Affiliated Hospital of Southern University of Science and Technology, Shenzhen 518020, China; 3 Guangdong Provincial Key Laboratory of Infection Immunity and Inflammation, Department of Pathogen Biology and Immunology, Medical School, Shenzhen University, Shenzhen 518060, China

**Keywords:** respiratory syncytial virus, critical illness, risk factor, predictive model

## Abstract

**Background/Objectives:** Respiratory syncytial virus (RSV) is a leading cause of hospitalization in children, but predictors of critical illness remain poorly defined. This study aimed to identify risk factors for critical RSV pneumonia and develop a predictive model. **Methods:** A retrospective analysis of 12,035 children hospitalized with RSV infection between 2019 and 2025 identified 304 eligible patients after applying exclusion criteria. Among these, 30 children with critical illness and 90 randomly selected non-critical controls were included. Clinical characteristics, laboratory parameters, and co-infection patterns were compared. Univariate, Lasso, and multivariable logistic regression analyses were performed to identify independent predictors, which were then incorporated into a nomogram. Model performance was assessed using the ROC curve, calibration plot, and decision curve analysis. **Results:** Among the 304 eligible children, 30 (9.9%) developed critical illness. Co-infection with three or more pathogens was most frequent in the critical group (43.3%), whereas single RSV infection predominated in the non-critical group (38.9%). Multivariable logistic regression identified four independent predictors of critical illness: interleukin-6 (IL-6), creatine kinase-MB (CK-MB), serum bilirubin excretion (SBE), and neutrophil percentage. The nomogram combining these factors exhibited excellent discrimination (AUC = 0.921, 95% CI: 0.868–0.974). The calibration curve closely matched the ideal 45° reference line (Hosmer–Lemeshow χ^2^ = 3.233, *p* = 0.919), and decision curve analysis demonstrated clinical benefit across threshold probabilities ranging from 0.01 to 0.99. **Conclusions:** Elevated IL-6, CK-MB, neutrophil percentage, and SBE are independent predictors of critical RSV infection in children. The nomogram based on these accessible biomarkers provides a robust tool for early risk assessment and guiding clinical decisions.

## 1. Introduction

Respiratory syncytial virus (RSV) remains the predominant viral pathogen responsible for acute lower respiratory tract infections in infants and young children worldwide, with approximately 33 million RSV-related cases each year in children under five years of age [1]. These infections lead to about 3.6 million hospital admissions and 118,200 deaths annually [2]. Although most infections are self-limiting and confined to the upper airways, a significant number of pediatric patients develop severe lower respiratory tract illness, particularly severe pneumonia [3,4]. This progression is characterized by profound respiratory distress, hypoxemia (SpO_2_ < 90%), central cyanosis, and the need for supplemental oxygen or mechanical ventilation, as defined by World Health Organization (WHO) standards. Children with viral bronchiolitis in early life are at increased risk of developing asthma later in childhood [3,4]. These serious cases are a major cause of hospitalization in young children, placing heavy demands on healthcare systems, families, and public health resources. Despite the known epidemiological significance, a major challenge remains in clinical settings: the inability to accurately predict, at the initial point of care, which children with RSV are most likely to progress to life-threatening pneumonia.

Currently, clinical decisions for children infected with RSV are typically based on a mix of established demographic risk factors—such as prematurity, very young age (particularly under six months), and existing congenital heart or chronic lung conditions—along with subjective evaluations of their respiratory condition [5]. However, these traditional risk stratifiers, while informative at a population level, lack the precision required for individual-level prognostication. Many children who develop severe pneumonia do not possess any of these classic high-risk features, while many who do have them experience only mild illness. This lack of precision creates two main clinical problems: the risk of under-triage, where a child who appears stable is sent home but then rapidly deteriorates unexpectedly, and the overuse of hospital resources by admitting and closely monitoring children who would have recovered without complications. The lack of a reliable, objective, and quantitative tool to forecast the critical progression from mild infection to severe pneumonia poses a major obstacle to providing timely, efficient, and personalized care.

The recent proliferation of electronic health records (EHRs), along with advancements in machine learning (ML) and statistical methods, provides a powerful approach to addressing this long-standing issue [6,7,8,9]. EHRs capture a rich, multidimensional dataset during a patient’s clinical encounter, including vital signs (e.g., respiratory rate, heart rate, and oxygen saturation), laboratory test results (e.g., white blood cell count, C-reactive protein), radiographic images, and detailed clinical observations [6]. Machine learning algorithms are uniquely suited to analyze these complex, high-dimensional data streams, uncovering non-linear relationships and subtle interaction effects between variables that are beyond the capacity of conventional statistical models or human intuition [7,10]. Statistical methods based on HER data have been widely applied to predict risk factors, such as predicting risk factors for severe *Mycoplasma pneumoniae* pneumonia and severe bacterial pneumonia [11], risk factors for severe *Chlamydia penumoniae* pneumonia in children [12], investigating the clinical characteristics of severe pneumonia caused by human bocavirus infection [13], and risk factors for severe lower respiratory tract infection among children under 5 years of age hospitalized with RSV [14] et al. However, risk factors specifically associated with RSV-induced critical pneumonia requiring interventions such as continuous positive airway pressure (CPAP), invasive mechanical ventilation (endotracheal intubation), or shock-necessitating vasoactive drugs have not yet been reported.

This study collected clinical data from patients hospitalized with RSV infection. Patients were divided into two groups—those with critical illness and those with non-critical illness—based on whether they needed CPAP, mechanical ventilation, or vasopressor drugs to treat shock. By analyzing the initial routine lab test results taken within 24 h of admission, this study aimed to identify risk factors linked to progression to critical illness and create a predictive model to recognize patients at high risk of developing severe RSV infection. The findings offer a theoretical basis for the clinical diagnosis and treatment of RSV infection.

## 2. Materials and Methods

### 2.1. Study Design and Data Collection

We carried out a single-center, retrospective cohort study involving children aged 17 days to 14 years who were admitted to Wuhan Children’s Hospital (affiliated with Tongji Medical College, Huazhong University of Science and Technology) with microbiologically confirmed RSV infections between 1 January 2019 and 31 May 2025. The RSV infection data for this study were obtained from the Scientific Research Data Platform of Wuhan Children’s Hospital, which compiles routine clinical information from various subsystems such as medical records, diagnoses, prescriptions, surgeries, laboratory tests, imaging, and nursing care. Only patients who had both a clinical diagnosis of pneumonia, bronchopneumonia, or sepsis and laboratory-confirmed RSV infection were included in the analysis. All analyses were based on laboratory test results collected within 24 h of hospital admission.

### 2.2. Patient Inclusion

Enrolled patients with RSV: (1) patients who tested positive for RSV; (2) patients with a clinical diagnosis of pneumonia, bronchopneumonia, or sepsis; (3) patients with complete clinical information.

Exclusion criteria for patients with RSV: (1) hospitalization due to respiratory infection within 30 days before admission; (2) the course of disease on admission was more than 15 days; (3) noro-virus or rotavirus infection (fecal nucleic acid test) or fungal infection; (4) use of immunoglobulin before admission; (5) underlying diseases: immunodeficiency disease, tumor, bronchopulmonary dysplasia, asthma, congenital heart disease with severe hemodynamic changes, cystic fibrosis, neuromuscular disease, hematologic disease, severe malnutrition, and other underlying diseases (immune throm-bocytopenia, favism disease, and so on); (6) admission to hospital because of other diseases.

**Figure 1:**
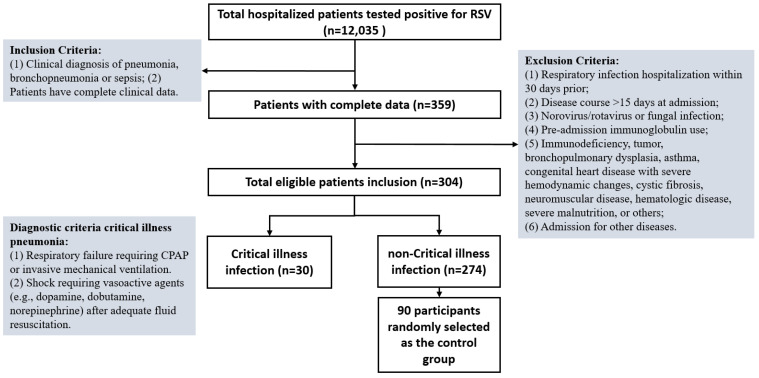
Patient inclusion flow chart.

Inclusion for critical illness group: (1) patients requiring continuous positive airway pressure (CPAP) or invasive mechanical ventilation (endotracheal intubation) due to respiratory failure; (2) presence of shock requiring vasoactive agents: after adequate fluid resuscitation, vasoactive drugs such as dopamine, dobutamine, and norepinephrine are still needed to maintain blood pressure and correct circulatory failure. The detailed process for screening cases is shown in Figure 1.

### 2.3. Statistical Analysis

Statistical analyses were conducted using R version 4.0.3, SPSS 25.0, and Prism 10.1.2 software. The Shapiro–Wilk test assessed the normality of the data distribution. Continuous variables with a normal distribution were reported as the mean ± standard deviation ($\bar{x}$ ± s), and differences between two groups were evaluated using the *t*-test. For continuous variables that were not normally distributed, comparisons were made using the Mann–Whitney U test, and results were presented as the median (Q1, Q3).

Risk factors for critical illness pneumonia with non-critical illness pneumonia were identified using logistic regression analysis, with the corresponding 95% CI and *p*-value. The analysis included a total of 120 eligible patients, of whom 30 had critical illness and 90 had non-critical illness. Because of the relatively small sample size, especially the limited number of critical cases (*n* = 30), the data were not divided into separate training and validation sets. Instead, all patients were utilized for both selecting variables and developing the model.

LASSO regression was employed for variable selection, with the optimal penalty parameter λ chosen as the value that minimized the cross-validated deviance via 10-fold cross-validation (λ_min_ = 0.060). The cross-validation was performed on the full set of 120 patients, and the selected variables were then used to build a multivariable logistic regression model. Based on the risk factors selected by LASSO regression, a multivariate logistic regression analysis was used to construct a predictive model. There were no missing values for any of the variables included in the LASSO and logistic regression analyses; therefore, no imputation method was required. The validation set served as an internal validation cohort to assess the model’s performance, and a nomogram was created to visually represent the model. Receiver operating characteristic (ROC) curves, calibration curves, and decision curve analysis (DCA) were plotted in both the training set and the validation set to verify the reliability of the model. A *p*-value < 0.05 was considered statistically significant.

## 3. Results

### 3.1. Description of Study Population

Between 1 January 2019 and 31 May 2025, a total of 12,035 hospitalized patients tested positive for RSV. After excluding patients with incomplete clinical information and those who did not meet the criteria for RSV infection, 304 eligible patients were included in this study (see Figure 1). Among these 304 patients, 30 cases (9.9%) were classified in the critical illness group, evenly split between males (15, 50%) and females (15, 50%). The remaining 274 cases (90.1%) were categorized as the non-critical illness group, consisting of 91 females (33.3%) and 183 males (66.7%). To ensure balanced data, 90 patients were randomly selected from the non-critical illness group to serve as controls. To assess potential selection bias, the 90 selected controls were compared with the remaining 184 non-critical patients; no significant differences were found in age, sex, comorbidities, or baseline laboratory results (all *p* > 0.05) (Table S1), confirming their representativeness. Additionally, there were no statistically significant differences between the critical and noncritical groups in terms of gender, BMI, or age (*p* > 0.05).

**Figure 2:**
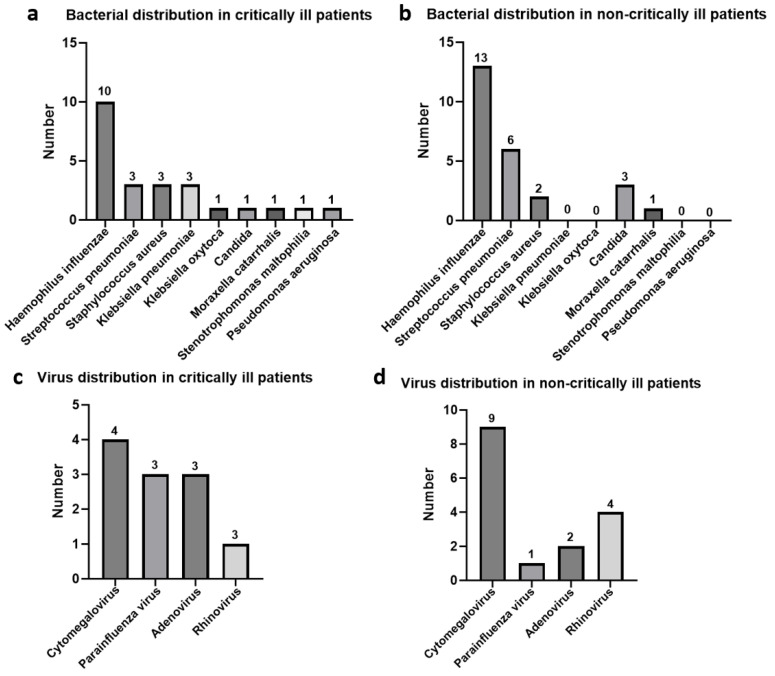
Bacteria and virus distribution in patients with co-infection. (**a**) Bacterial distribution in critically ill patients. (**b**) Bacterial distribution in non-critically ill patients. (**c**) Virus distribution in critically ill patients. (**d**) Virus distribution in non-critically ill patients.

In the group with critical illness, co-infection involving three or more pathogenic types was the most prevalent, making up 43.3% (Table 1). In contrast, in the non-critical illness group, a single RSV infection was the most common, accounting for 38.89% (Table 1). Among cases with bacterial co-infections, *Haemophilus influenzae* was the predominant pathogen in both critical and non-critical groups (Figure 2a,b). For viral co-infections, Cytomegalovirus was the most frequently observed in both groups (Figure 2c,d). These results suggest that co-infection with three or more types of pathogens may contribute to critical illness in pediatric patients, with *Haemophilus influenzae* and *Cytomegalovirus* being the most common bacteria and virus co-infecting with RSV in these cases.

### 3.2. A Comparison of Laboratory Test Results

Analysis of laboratory test results collected within 24 h of hospital admission revealed that the critical illness group had lower levels of APTT, IL-10, and lymphocyte percentage compared to the non-critical illness group (*p* < 0.05) (Table 2). Conversely, IL-6, neutrophil percentage, and CK-MB were significantly higher in the critical illness group than in the non-critical illness group (*p* < 0.05) (Table 2). These findings suggest that critically ill patients experience multiple pathological changes, including an excessive inflammatory response (IL-6 ↑) coupled with a reduced anti-inflammatory response (IL-10 ↓), immunosuppression (lymphocyte ↓, neutrophil ↑), a hypercoagulable state (APTT ↓), and myocardial injury (CK-MB ↑).

### 3.3. Risk Factors for Critical Illness Infection Outcomes

Clinical data from the two groups were compared using stepwise logistic regression. Initially, univariate logistic regression analysis was conducted (see Table 3). The findings revealed that co-infection with three or more pathogenic types, PaCO_2_, SBE, IL-6, hemoglobin levels, lymphocyte percentage, and neutrophil percentage were independent risk factors associated with the critically ill group.

**Figure 3:**
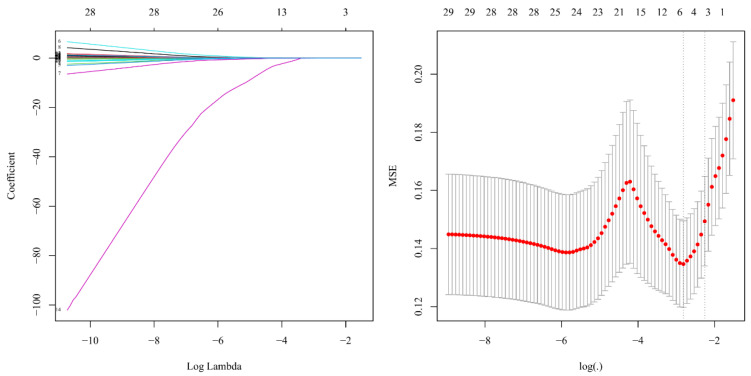
Lasso regression to screen risk factors for predicting critical illness. (**Left**) LASSO coefficient profiles of the variables. The colorful lines represent the LASSO coefficient paths of individual predictors across log(λ). Each color corresponds to a different variable; (**Right**) the best penalty coefficient lambda was selected using a tenfold cross-validation and minimization criterion. The red dot marks the log(λ) that gives the minimum mean squared error (MSE). The left dashed line represents the log(λ) corresponding to the minimum MSE. The right dashed line represents the log(λ) where the MSE equals the minimum MSE plus one standard error.

To ensure that no important variables were overlooked, a relatively lenient significance threshold was applied during the univariate analysis to identify potential candidate variables. These candidates were then entered simultaneously into Lasso regression and multivariable logistic regression analyses to address multicollinearity and enhance the predictive model (Figure 3 and Table 4). The findings indicated that IL-6 (OR = 1.027, 95% CI: 1.011–1.044, *p* < 0.001), CK-MB (OR = 1.030, 95% CI: 1.007–1.055, *p* = 0.013), SBE (OR = 1.246, 95% CI: 1.010–1.537, *p* = 0.040), and neutrophil percentage (OR = 1.082, 95% CI: 1.041–1.125, *p* < 0.001) were independent predictors of critical illness (Table 4). This implies that for every one-unit increase in IL-6 (e.g., 1 pg/mL), the odds of developing critical illness rise by about 2.7% (since 1.027 − 1 = 0.027), assuming other factors remain constant. The 95% confidence interval (1.011–1.044) excludes 1, confirming the result is statistically significant at the *p* < 0.05 level. Likewise, each unit increase in CK-MB (OR = 1.030) is associated with a 3.0% increase in odds; each unit increase in SBE (OR = 1.246) corresponds to a 24.6% increase; and each percentage point increase in neutrophil percentage (OR = 1.082) relates to an 8.2% increase in the odds of critical illness.

### 3.4. Construction of a Prediction Nomogram for Critical Illness Infection

**Figure 4:**
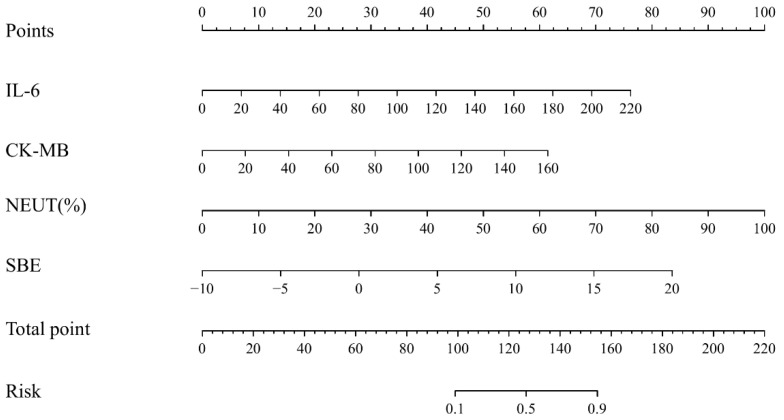
Nomogram for the risk of critical illness. CK-MB, creatine kinase isoenzyme; NEUT, neutrophil percentage; SBE, standard base excess.

Based on the four independent risk factors identified by multivariable logistic regression analysis, a visualized nomogram for predicting the probability of critical illness was constructed, as illustrated in Figure 4. This model incorporates four variables: IL-6, CK-MB, SBE, and neutrophil percentage. The nomogram for predicting critical illness occurrence in children with RSV infection consists of four variable axes (IL-6, CK-MB, SBE, and neutrophil percentage), one point axis (points), one total point axis (total points), and a corresponding risk axis (risk), as shown in Figure 4. When the sum of the scores of the factors is 127, the corresponding probability of developing severe illness is 50%.

### 3.5. Internal Validation of the Nomogram Prediction Model

**Figure 5:**
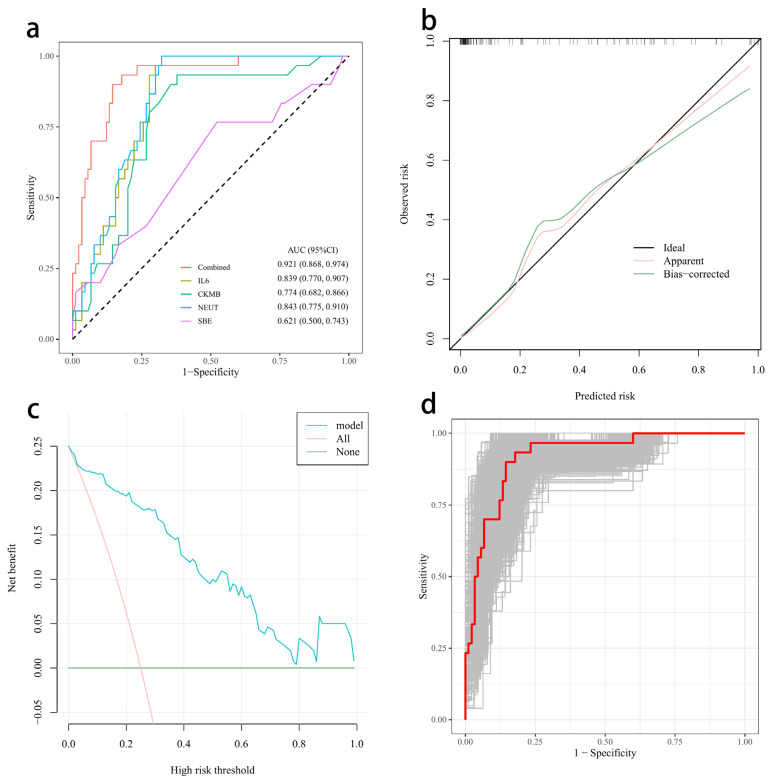
The predictive value of IL-6, CK-MB, neutrophil percentage, and SBE in critical illness infection. (**a**) ROC curve of different risk factors. The AUC of IL-6 in predicting critical illness infection was 0.839 (0.770, 0.907). The AUC of CK-MB was 0.774 (0.682, 0.866). The AUC of neutrophil percentage was 0.843 (0.775, 0.910). The AUC of SBE was 0.621 (0.5, 0.743). The area under the ROC curve of IL-6 combined with CK-MB, neutrophil percentage, and SBE for predicting critical illness was 0.921 (0.868, 0.974). The diagonal dashed line indicates the reference line of random classification (AUC = 0.5). (**b**) Calibration curve. (**c**) DCA curve. (**d**) Bootstrap ROC curve. ROC curve, receiver operating characteristic curve; AUC, area under the curve. Gray lines indicate ROC curves from bootstrap resamples.

Subsequently, we used the risk factors to analyze the predictive value for critical illness infection using receiver operating characteristic (ROC) curve analysis. The findings presented in Figure 5a revealed: (1) The area under the ROC curve (AUC) for IL-6 in predicting critical illness infection was 0.839 (95% CI: 0.770, 0.907). (2) The AUC for CK-MB was 0.774 (95% CI: 0.682, 0.866). (3) The AUC for neutrophil percentage was 0.843 (95% CI: 0.775, 0.910). (4) The AUC for SBE was 0.621 (95% CI: 0.5, 0.743). (5) The area under the ROC curve for IL-6 combined with CK-MB, neutrophil percentage, and SBE for predicting critical illness was 0.921 (95% CI: 0.868, 0.974) (Figure 5a). These ROC curve results demonstrate that the model possesses strong predictive capability.

Figure 5b displays the calibration curve of the prediction model. This curve closely follows the 45-degree reference line, suggesting a strong alignment between the predicted risk of severe illness and the actual observed cases. The Hosmer–Lemeshow test further confirmed a good fit of the model (χ^2^ = 3.233, *p* = 0.919 > 0.05). The results of the decision curve analysis (DCA) are shown in Figure 5c. The DCA plot demonstrates that the model provides greater clinical benefit across a threshold probability range of 0.01 to 0.99. Figure 5d displays a series of ROC curves generated from 1000 bootstrap resampling iterations, which closely match the original ROC curve, reinforcing the model’s reliable discriminative performance. Overall, these findings suggest that the model holds significant potential for clinical use.

## 4. Discussion

This study marks a notable breakthrough in the early detection of pediatric patients with RSV infection who are at high risk of developing severe pneumonia. Researchers developed and validated a predictive nomogram using four easily accessible laboratory biomarkers measured upon admission: IL-6, CK-MB, neutrophil percentage, and SBE. This tool provides frontline healthcare providers with a practical and effective means of assessment. The model demonstrated excellent accuracy, with an area under the receiver operating characteristic curve of 0.921, along with strong calibration and proven clinical usefulness through decision curve analysis. These findings highlight its potential to improve clinical practice by facilitating timely, risk-based treatment decisions.

The biological relevance of the identified predictors offers a convincing explanation for the underlying mechanisms of severe RSV disease. IL-6 plays a central role as a key independent predictor, aligning with extensive research that links an uncontrolled, excessive inflammatory response by the host as the main cause of serious lower respiratory tract infections in infants [15,16,17,18]. High levels of IL-6 indicate a strong pro-inflammatory reaction that can result in increased blood vessel permeability, lung swelling, and, eventually, respiratory failure [19,20]. This supports the idea that the severity of RSV illness is often driven more by the host’s immune system overreacting than by the direct damage caused by the virus itself. Likewise, an elevated neutrophil percentage is a well-known blood marker of acute systemic inflammation, further supporting this inflammatory explanation. The finding of a significantly reduced lymphocyte percentage in the critically ill group suggests relative lymphopenia, which could reflect either the trapping of lymphocytes in inflamed tissues or a form of immune suppression that accompanies the intense inflammatory response, a pattern seen in other serious infections [21,22,23,24]. We also address the concern that the neutrophil percentage in our non-severe group (39.89 ± 20.27%) might appear low relative to the Japanese adult range of 50–70%. This is not due to inter-laboratory differences. Our study population was children, in whom the normal neutrophil percentage is 20–55% (our laboratory reference range). The non-severe group values lie within this pediatric range, while the severe group values (65.12 ± 12.32%) are markedly elevated above the pediatric range, indicating severe inflammation. Thus, the observed differences are clinically meaningful and reflect age- and disease-specific physiology, not laboratory error.

We identified that CK-MB was a strong independent predictor of RSV-induced critical illness. CK-MB was not significant in univariate analysis but became significant after multivariate adjustment. This is primarily explained by confounding: the unadjusted effect of CK-MB was masked by covariates such as age, sex, and comorbidities, and only after controlling for these factors did its independent association with critical illness emerge. Additionally, the limited sample size (especially the small number of critical cases) may have reduced the power to detect a borderline effect in the univariate analysis, whereas multivariate adjustment reduced residual variance and improved precision. Therefore, the multivariate model likely provides a more accurate estimate of the true effect of CK-MB.

Traditionally, CK-MB was viewed as a specific marker for myocardial injury. However, recent studies have shown that increased levels of CK-MB are strongly linked to severe illness and higher mortality rates in patients with COVID-19 [25], tuberculosis cases [26], and cases of rhabdomyolysis induced by influenza A and RSV [27]. Despite this, there have been no reports specifically connecting CK-MB to severe infections caused by RSV. Severe RSV bronchiolitis can significantly strain the cardiovascular system through various mechanisms, such as low oxygen levels leading to heart muscle ischemia, acidosis, and the direct harmful effects of inflammatory cytokines like IL-6 and TNF-α [28,29,30,31,32]. Therefore, elevated CK-MB levels might serve as an early warning sign of this combined heart and lung dysfunction, suggesting that critical illness progression involves not only lung damage but also failure of the integrated heart–lung system. This understanding expands the clinical view of severe RSV from being solely a lung disease to a systemic condition with important cardiac consequences.

Identifying SBE as a predictor further enhances this comprehensive perspective. Base excess (BE) is a dependable indicator for evaluating acid–base imbalances, unaffected by respiratory influences, and accurately reflects the true metabolic acid–base status [33]. SBE, also known as extracellular fluid BE, is used to differentiate blood BE, with the Van Slyke formula-based SBE calculation recommended as the preferred method in clinical settings due to its better representation of in vivo conditions compared to BE [33,34]. Both excessively high and low levels of SBE are associated with an increased risk of acute kidney injury in ICU patients [35]. A more negative SBE indicates a greater level of metabolic acidosis, which in critically ill children usually results from tissue hypoperfusion and anaerobic metabolism caused by shock or severe respiratory failure. Including SBE in the model emphasizes that the severity of RSV is closely connected to the extent of end-organ perfusion and metabolic imbalance, offering vital insight into the patient’s overall physiological resilience and stability.

The strong association between polymicrobial co-infection (three or more pathogens) and critical illness is another key finding. In univariate analysis, co-infection with three or more pathogens was associated with critical illness (OR = 4.063, *p* = 0.012). However, this variable was not selected in the LASSO procedure and did not enter the final multivariate model. This does not mean that co-infection has no predictive value at all. This may be due to limited statistical power (only 30 critical cases) or confounding by other clinical factors. Future studies with larger sample sizes are warranted to further clarify the independent impact of co-infection on the prediction of critical illness outcomes. Although RSV is the main pathogen, the presence of additional viral (e.g., *Cytomegalovirus*) or bacterial (e.g., *Haemophilus influenzae*) agents seems to significantly worsen the disease, consistent with previous research [36,37]. This may be due to synergistic effects that increase epithelial damage [38], impair mucociliary clearance [39,40], or further disrupt the host immune response [41], creating a perfect storm for rapid clinical deterioration.

However, this study has several limitations. Firstly, the retrospective, single-center design introduces the potential for selection bias and limits the generalizability of the model, making it difficult to apply to hospitals with different levels of care in other regions. Secondly, the relatively small number of critical cases (*n* = 30) may have constrained the model’s ability to capture the full spectrum of risk factors, particularly for rare but high-impact variables. Thirdly, several clinically important predictors of RSV severity—including prematurity, respiratory rate, oxygen requirement at presentation, nutritional status, and radiographic findings—were not available in our retrospective database. Their omission limits the model’s applicability in real-world clinical settings, where such bedside parameters are routinely used. Future studies should prospectively collect these variables to improve model performance and generalizability. At last, only internal bootstrap validation was performed, with no external validation. To address these limitations, future multicenter, prospective studies with larger sample sizes of severe cases are warranted, along with the inclusion of clinical parameters such as respiratory rate and radiographic findings to optimize the nomogram model. External validation should be conducted to verify the model’s applicability across different regions and age groups. Furthermore, the specific molecular mechanisms linking CK-MB and SBE to severe RSV infection should be further explored to identify new targets for clinical intervention.

In summary, this study effectively transforms intricate clinical and laboratory information into an easy-to-understand, visual, and precise predictive model. The nomogram, which relies on biologically relevant indicators of inflammation, heart stress, and metabolic issues, provides a clear method to enhance the care of children with RSV. Future studies should focus on prospective, multicenter validation and incorporating the tool into electronic health records for real-time risk assessment, aiming to decrease illness and death rates in this at-risk group.

## Figures and Tables

**Table 1:** Demographic characteristics between the critical illness and non-critical illness groups.

Characteristics	Critical Illness (*n* = 30)	Non-Critical Illness (*n* = 90)	t/χ^2^/z	*p*
Age (month)	8.61 (3.75, 24.86)	16.84 (4.88, 33.67)	−1.349	0.177
Sex			100.800	0.349
male	15 (50.00)	60 (66.67)		
female	15 (50.00)	30 (33.33)		
BW (kg)	8.50 (5.22, 11.50)	11.00 (7.50, 14.00)	−2.096	0.036
HT (cm)	69.50 (60.00, 86.25)	81.50 (65.75, 97.00)	−1.861	0.063
BMI	16.34 (14.87, 17.35)	15.69 (14.72, 17.71)	−0.288	0.773
Co-infection			194.255	0.441
Single RSV infection	7 (23.3%)	35 (38.9%)		
Bacterial co-infection	6 (20%)	13 (14.4%)		
Viral co-infection	3 (10%)	16 (17.8%)		
Mycoplasma	1 (3.3%)	10 (11.1%)		
Three or more pathogenic types co-infection	13 (43.3%)	16 (17.8%)		

BW, body weight; HT, height; BMI, body mass index. The percentages add up to 99.9% instead of 100% due to rounding.

**Table 2:** Comparison of laboratory tests between the critical illness and non-critical illness groups.

Characteristics	Critical Illness (*n* = 30)	Non-Critical Illness (*n* = 90)	t/χ^2^/z	*p*
APTT (s)	29.80 (26.95, 34.60)	33.65 (29.20, 37.62)	−2.246	**0.025**
PT (s)	11.20 (10.50, 12.60)	11.35 (10.60, 12.30)	−0.382	0.702
Fibrinogen (g/L)	2.88 ± 1.21	3.13 ± 1.17	−1.005	0.317
D-dimer (mg/L)	0.54 (0.32, 1.20)	0.42 (0.28, 0.64)	−1.791	0.073
hs-CRP (mg/L)	9.55 (2.43, 23.93)	9.65 (2.39, 29.30)	−0.076	0.940
SpO_2_ (%)	91.00 (87.00, 96.00)	93.00 (91.00, 96.00)	−1.851	0.064
PaCO_2_ (mmHg)	38.50 (33.00, 45.75)	36.00 (31.00, 40.25)	−1.935	0.053
PaO_2_ (mmHg)	63.50 (54.00, 81.00)	65.50 (60.00, 76.50)	−1.071	0.284
SBE (mmol/L)	0.00 (−0.75, 2.00)	0.00 (−3.08, 1.00)	−2.014	**0.044**
pH	7.38 (7.33, 7.45)	7.41 (7.37, 7.43)	−1.291	0.197
IL-6 (pg/mL)	42.45 (21.71, 91.28)	12.10 (8.15, 25.20)	−5.545	**<0.001**
IL-10 (pg/mL)	8.25 (5.95, 11.96)	13.35 (7.51, 22.84)	−2.658	**0.008**
TNF-α (pg/mL)	3.49 (2.86, 6.25)	3.33 (2.20, 5.36)	−1.015	0.310
Hb (g/L)	117.00 (104.25, 125.00)	114.00 (104.00, 121.00)	−1.258	0.208
WBC (10^9^/L)	7.53 (5.76, 11.81)	8.61 (6.62, 11.39)	−0.864	0.388
PLT (10^9^/L)	397.77 ± 196.88	340.01 ± 133.20	1.810	0.073
Lymphocyte Percentage (%)	36.66 ± 18.41	46.90 ± 21.68	−2.323	**0.022**
Neutrophil Percentage (%)	65.12 ± 12.32	39.89 ± 20.27	6.422	**<0.001**
CK (U/L)	104.00 (58.75, 147.25)	82.00 (59.00, 119.50)	−1.267	0.205
CK-MB (U/L)	44.65 (35.00, 79.00)	28.00 (23.00, 38.50)	−4.477	**<0.001**
ALT (U/L)	20.50 (13.75, 29.75)	18.00 (11.75, 32.00)	−0.995	0.320
AST (U/L)	44.50 (29.50, 54.25)	40.50 (32.75, 55.00)	−0.297	0.766
TBIL (μmol/L)	6.80 (3.48, 8.20)	4.90 (3.50, 7.60)	−1.121	0.262
Creatinine (μmol/L)	22.00 (18.95, 27.02)	22.60 (19.17, 28.80)	−0.485	0.628
BUN (mmol/L)	3.00 (2.18, 3.75)	2.96 (2.39, 3.73)	−0.100	0.920
PCT (ng/mL)	0.20 (0.11, 0.35)	0.18 (0.11, 0.50)	−0.055	0.956

APTT, activated partial thromboplastin time; PT, prothrombin time; hs-CRP, high-sensitivity C-reactive protein; PaCO_2_, partial pressure of carbon dioxide; PaO_2_, partial pressure of oxygen; SBE, standard base excess; Hb, hemoglobin concentration; WBC, white blood cell count; PLT, platelet count; CK, creatine kinase; CK-MB, creatine kinase isoenzyme; ALT, alanine aminotransferase; AST, aspartate aminotransferase; TBIL, total bilirubin; BUN, blood urea nitrogen; PCT, procalcitonin; SpO_2_, peripheral capillary oxygen saturation. Bold font indicates *p* < 0.05.

**Table 3:** Univariate logistic regression was used to analyze the risk factors for critical illness group.

Characteristics	RC	SE	z	OR (95% CI)	*p*
Age	0.003	0.009	0.294	1.003 (0.985, 1.021)	0.769
Sex (female)	−0.693	0.428	−1.619	0.500 (0.216, 1.157)	0.105
BW	0.025	0.04	0.618	1.025 (0.948, 1.109)	0.537
HT	0.013	0.011	1.217	1.013 (0.991, 1.035)	0.224
BMI	0.037	0.086	0.427	1.038 (0.877, 1.228)	0.669
Single RSV infection	-	-	-	-	-
Two pathogenic types co-infection	0.248	0.545	0.456	1.281 (0.440, 3.729)	0.648
Three or more pathogenic types co-infection	1.402	0.558	2.514	4.063 (1.361, 12.130)	**0.012**
APTT	0.05	0.034	1.446	1.051 (0.983, 1.124)	0.148
PT	0.008	0.154	0.050	1.008 (0.745, 1.363)	0.960
Fibrinogen	0.191	0.19	1.004	1.210 (0.834, 1.757)	0.315
D-dimer	0.04	0.095	0.421	1.041 (0.864, 1.254)	0.674
hs-CRP	0.005	0.008	0.622	1.005 (0.989, 1.021)	0.534
SpO_2_	0.008	0.05	0.150	1.008 (0.914, 1.112)	0.880
PaCO_2_	−0.048	0.023	−2.038	0.953 (0.911, 0.997)	**0.042**
PaO_2_	0.023	0.015	1.518	1.023 (0.994, 1.054)	0.129
SBE	−0.136	0.06	−2.243	0.873 (0.776, 0.982)	**0.025**
pH	5.305	3.362	1.578	201.341 (0.277, 146,461.811)	0.115
IL-6	0.107	0.033	3.237	1.113 (1.043, 1.187)	**0.001**
IL-10	0.038	0.023	1.654	1.039 (0.993, 1.087)	0.098
TNF-α	0.002	0.004	0.446	1.002 (0.994, 1.010)	0.655
Hb	0.075	0.018	4.182	1.078 (1.041, 1.117)	**<0.001**
WBC	0.012	0.037	0.334	1.012 (0.941, 1.088)	0.738
PLT	−0.002	0.001	−1.772	0.998 (0.996, 1.000)	0.076
Lymphocyte percentage	0.024	0.011	2.245	1.024 (1.002, 1.047)	**0.025**
Neutrophil percentage	0.025	0.011	2.394	1.025 (1.003, 1.048)	**0.017**
CK	−0.003	0.002	−1.678	0.997 (0.993, 1.001)	0.093
CK-MB	−0.005	0.008	−0.716	0.995 (0.980, 1.011)	0.474
ALT	0.005	0.009	0.493	1.005 (0.987, 1.023)	0.622
AST	−0.001	0.008	−0.147	0.999 (0.983, 1.015)	0.883
TBIL	−0.007	0.029	−0.229	0.993 (0.938, 1.051)	0.819
Creatinine	0.013	0.031	0.425	1.013 (0.953, 1.077)	0.671
BUN	−0.05	0.177	−0.282	0.951 (0.672, 1.346)	0.778
PCT	0.12	0.191	0.630	1.127 (0.775, 1.639)	0.529

BW, body weight; HT, height; BMI, body mass index; APTT, activated partial thromboplastin time; PT, prothrombin time; hs-CRP, high-sensitivity C-reactive protein; PaCO_2_, partial pressure of carbon dioxide; PaO_2_, partial pressure of oxygen; SBE, standard base excess; Hb, hemoglobin concentration; WBC, white blood cell count; PLT, platelet count; CK, creatine kinase; CK-MB, creatine kinase isoenzyme; ALT, alanine aminotransferase; AST, aspartate aminotransferase; TBIL, total bilirubin; BUN, blood urea nitrogen; PCT, procalcitonin; SpO_2_, peripheral capillary oxygen saturation. Bold font indicates *p* < 0.05.

**Table 4:** Multivariate logistic regression to predict risk factors for critical illness.

Characteristics	RC	SE	z	OR (95% CI)	*p*
Constant term	−7.818	1.548	−5.050	0.000 (0.000, 0.008)	<0.001
Neutrophil percentage	0.079	0.02	4.049	1.082 (1.041, 1.125)	<0.001
IL-6	0.027	0.008	3.541	1.027 (1.011, 1.044)	<0.001
CK-MB	0.03	0.012	2.492	1.030 (1.007, 1.055)	0.013
SBE	0.22	0.107	2.051	1.246 (1.010, 1.537)	0.040

CK-MB, creatine kinase isoenzyme; SBE, standard base excess.

## Data Availability

The data presented in this study are openly available in [4TU] at [doi: 10.5074/4b1bb8ea-efa2-4f02-95b6-db62136222e1], reference number [42].

## Supplementary Materials

The following supporting information can be downloaded at https://www.mdpi.com/article/10.3390/diagnostics16111701/s1: Table S1. Demographic characteristics between the selected and unselected controls.

## Abbreviations

RSV	Respiratory Syncytial Virus
ROC	Receiver Operating Characteristic
DCA	Decision Curve Analysis
APTT	Activated Partial Thromboplastin Time
PT	Prothrombin Time
hs-CRP	High-sensitivity C-reactive Protein
PaCO_2_	Partial Pressure of Carbon Dioxide
PaO_2_	Partial Pressure of Oxygen
SBE	Standard Base Excess
Hb	Hemoglobin Concentration
WBC	White Blood Cell Count
PLT	Platelet Count
CK	Creatine Kinase
CK-MB	Creatine Kinase Isoenzyme
ALT	Alanine Aminotransferase
AST	Aspartate Aminotransferase
TBIL	Total Bilirubin
BUN	Blood Urea Nitrogen
PCT	Procalcitonin
SpO_2_	Peripheral Capillary Oxygen Saturation
CPAP	Continuous Positive Airway Pressure
RC	Regression Coefficient
SE	Standard Error
