# Supplementary material for: Predictive Model for Critical Illness Infection in Hospitalized Children with RSV Infection: A Retrospective Study
Source: Diagnostics (Basel). 2026 May 31;16(11):1701. doi: 10.3390/diagnostics16111701 (PMC13256771; doi:10.3390/diagnostics16111701)
Supplement: Supplementary file 1 [file diagnostics-16-01701-s001.zip › diagnostics-4316907-supplementary.pdf]

Table S1 Demographic characteristics between the selected and unselected controls.

Characteristics	Selected controls (n=90)	Unselected controls (n=184)	Mann-Whitney U statistic	Mann-Whitney z statistic	p
Age (month)	16.835(4.9,33.7)	16.525(6.3,30.9)	8059.500	-0.358	0.720
Sex	2.000(1.0,2.0)	2.000(1.0,2.0)	8175.000	-0.210	0.834
male	60(66.67)	125(67.93)			
female	30(33.33)	59(32.07)			
BW (kg)	11.000(7.5,14.0)	10.000(8.0,13.0)	7896.500	-0.552	0.581
HT (cm)	81.500(65.8,97.0)	80.000(68.0,92.0)	8079.500	-0.254	0.800
BMI	15.690(14.7,17.7)	16.298(14.9,18.0)	7841.000	-0.642	0.521
APTT(s)	33.650(29.2,37.6)	32.250(28.8,37.9)	7775.500	-0.819	0.413
PT (s)	11.350(10.6,12.3)	11.200(10.6,12.0)	7752.000	-0.858	0.391
Fibrinogen (g/L)	3.040(2.1,3.8)	2.770(2.2,3.7)	7648.000	-1.026	0.305
D-dimer (mg/L)	0.425(0.3,0.6)	0.390(0.3,0.5)	5885.000	-0.668	0.504
hs-CRP (mg/l)	9.650(2.4,29.3)	7.620(2.5,27.5)	6280.000	-0.131	0.896
SpO_2 (%)	93.000(91.0,96.0)	93.000(91.0,95.0)	8242.500	-0.061	0.951
$PaCO_2$ (mmHg)	36.000(31.0,40.3)	36.000(32.0,40.0)	7982.500	-0.483	0.629
PaO_2 (mmHg)	65.500(60.0,76.5)	67.500(60.3,76.0)	8097.000	-0.297	0.766
SBE (mmol/L)	0.000(-3.1,1.0)	-1.000(-4.0,1.0)	6301.500	-0.615	0.539
pH	7.410(7.4,7.4)	7.400(7.4,7.4)	7947.000	-0.542	0.588
IL-6 (pg/ml)	12.100(8.1,25.2)	15.740(9.0,33.2)	7399.000	-1.430	0.153
IL-10 (pg/ml)	13.345(7.5,22.8)	10.580(6.9,26.0)	7966.500	-0.509	0.611
TNF-α(pg/ml)	3.325(2.2,5.4)	3.785(2.7,6.7)	7086.500	-1.937	0.053
Hb (g/L)	114.000(104.0,121.0)	114.000(106.0,122.0)	7683.000	-0.969	0.332
WBC (10^9/L)	8.615(6.6,11.4)	8.290(6.0,10.9)	7601.500	-1.101	0.271
PLT (10^9/L)	323.000(260.3,434.5)	332.000(241.8,426.8)	8131.500	-0.241	0.810
Lymphocyte Percentage (%)	47.950(30.7,63.5)	45.600(33.2,61.7)	8267.500	-0.020	0.984

Neutrophil Percentage (%)	37.800(23.7,55.3)	44.000(27.3,55.4)	7420.000	-1.396	0.163
CK (U/L)	82.000(59.0,119.5)	87.000(59.0,136.0)	7347.500	-1.175	0.240
CK-MB (U/L)	28.000(23.0,38.5)	30.000(23.0,42.0)	7217.000	-1.392	0.164
ALT (U/L)	18.000(11.8,32.0)	16.500(12.3,24.0)	8111.000	-0.275	0.784
AST (U/L)	40.500(32.8,55.0)	42.000(33.0,49.8)	8251.000	-0.047	0.962
TBIL (μmol/L)	4.900(3.5,7.6)	4.950(3.5,7.0)	7942.000	-0.549	0.583
Creatinine (μmol/L)	22.600(19.2,28.8)	23.500(20.3,27.8)	7477.500	-1.303	0.193
BUN (mmol/L)	2.965(2.4,3.7)	3.000(2.2,3.9)	8127.000	-0.248	0.804
PCT (ng/ml)	0.185(0.1,0.5)	0.150(0.1,0.4)	7301.500	-1.589	0.112

BW, Body Weight; HT, Height; BMI, Body Mass Index. APTT, Activated Partial Thromboplastin Time; PT, Prothrombin Time; hs-CRP, High-sensitivity C-reactive Protein; $PaCO_2$, Partial Pressure of Carbon Dioxide; PaO_2, Partial Pressure of Oxygen; SBE, Standard Base Excess; Hb, Hemoglobin Concentration; WBC, White Blood Cell Count; PLT, Platelet Count; CK, Creatine Kinase; CK-MB, Creatine Kinase Isoenzyme; ALT, Alanine Aminotransferase; AST, Aspartate Aminotransferase; TBIL, Total Bilirubin; BUN, Blood Urea Nitrogen; PCT, Procalcitonin; SpO_2, Peripheral capillary oxygen saturation.
